# Temporal Trends in Fertility Rates: A Nationwide Registry Based Study from 1901 to 2014

**DOI:** 10.1371/journal.pone.0143722

**Published:** 2015-12-02

**Authors:** Martin Blomberg Jensen, Lærke Priskorn, Tina Kold Jensen, Anders Juul, Niels Erik Skakkebaek

**Affiliations:** 1 Department of Oral Medicine, Infection and Immunity, Harvard School of Dental Medicine, Boston, MA, United States of America; 2 Department of Growth and Reproduction and International Research and Research Training Centre in Endocrine Disruption of Male Reproduction and Child Health (EDMaRC), Rigshospitalet, Faculty of Medical and Health Sciences, University of Copenhagen, Blegdamsvej 9, DK-2100 Copenhagen, Denmark; University of Science and Technology of China, CHINA

## Abstract

**Objective:**

Increasing age at first childbirth has been suggested to increase the risk for infertility. Our objective is to determine whether women above thirty years of age historically have been able to sustain fertility rates above replacement level.

**Design:**

A descriptive nationwide Danish study using birth registries from 1901–2014.

**Setting:**

Information on women’s age at childbirth was obtained by using records from primary, secondary and tertiary institutions.

**Participants:**

Mothers to 8,024,969 live births.

**Main outcome measures:**

Mothers were stratified according to age at childbirth to determine total and age specific fertility rates.

**Results:**

Total fertility rate (TFR) decreased from 4.1 to 1.8 children per woman and age specific fertility also decreased from 1901 to 2014. Women aged 30–34, 35–39 or 40–44 years in the first decade of the 20th century had higher fertility rates than the corresponding five year younger age groups (25–29, 30–34 and 35–39, respectively) have had for the last 65 years. On average, women gave birth to two children after the age of 30 and one or more child after 35 years of age in the beginning of the 1900s. Furthermore, women more than 40 years of age accounted for 10% of TFR in 1901 compared with 4% in 2014 despite usage of assisted reproduction.

**Conclusion:**

This nationwide study shows that women above 30 years of age historically have been able to sustain fertility rates above replacement level. This implies that other factors besides age are strong determinants of fertility in women above 30 years of age.

## Introduction

Age is a determining factor for fertility and fecundity in women, and fertility decreases with age. Some clinicians have indicated that increasing maternal age for the first childbirth may lead to increased risk of infertility for women, although few studies have investigated this on the population level [1–6].

Historically, female reproduction has focussed on two clinical problems: to delay childbearing and cure infertility. Effective contraception was introduced in the 1960s and solved the first problem, while infertility was remedied by the development of assisted reproductive techniques (ART) in the following decades [1]. The massive use of contraception and concomitant social, behavioural and demographic changes increased the tendency for many women to delay childbirth in the developed world [2,3]. The clinical concern has been that advancing female age has clinical consequences [4,5]. When a woman’s chronological age exceeds 35 years, the oocytes more frequently have genetic or structural abnormalities that subsequently impose a decline in the rates of implantation, pregnancies and live birth rates [3,6]. Hence, the solution of using contraceptives to solve one problem might have intensified the magnitude of the infertility problem by delaying the first childbirth. Fertility can be defined as the natural capability to produce offspring and indeed, fertility rates have decreased in the recent century. However, these changes are mainly the result of socioeconomic and lifestyle changes rather than changes in biological ability to conceive (fecundity). Fertility rates are influenced by multiple factors, but the concern has been that increasing maternal age for the first childbirth may lead to an increased risk of infertility.

The prevalence of infertility ranges from 7–26% of couples in the childbearing age in the developed countries [7,8]. Fortunately, ART is effective for many couples even in women above the age of 40 [9]. Low fertility rates and increased infertility are not only a burden for the patients but also for the society due to the resultant demographic changes and the immediate economic burden [8]. A relevant question is whether an upper maternal age limit to sustain fertility rates at replacement level is nearly approaching due to the increased maternal age for first childbirth. The isolated impact of age on fertility is difficult to investigate especially retrospectively, but in infertile couples, several studies have shown that chronological age is an important predictor of ovarian response to stimulating hormone therapy and live birth rate [1,3]. Important knowledge has been gained from oocyte retrieval during ART indicating that a combination of environmental and genetic factors contributes to the decrease and reduced quality of the oocyte pool with age [1,6]. Historical data from married couples clearly demonstrated that chronological age predicted the chance of live births and found a large decrease in fertility after 35–40 years of age [10]. Increasing maternal age at first childbirth has been observed in the 1980s in both France and Denmark simultaneously with increased fertility rates [10,11]. This indicates that these observations are not mutually exclusive. The onset of the accelerated fertility loss with age is controversial and further complicated by the fact that biological age and chronological ovarian age are not always in accord. Therefore, the use of biomarkers as predictors of the antral follicle count such as serum concentrations of anti-Müllerian hormone (AMH), follicle stimulating hormone (FSH), Inhibins and imaging of the ovary are increasingly used clinically to determine whether the woman has an expected oocyte pool or accelerated biological ovarian ageing [1,12,13]. In this study, total and age specific fertility rates were evaluated nationwide in Danish women giving birth from 1901 to 2014. To determine whether women above 30 years of age on the population level have been able to sustain fertility rates at replacement level historically.

## Materials and Methods

This study population comprises all live births in Denmark from 1901 to 2014 (n = 8,024,969), and the women’s age at childbirth. For women giving birth after 1960, information on age at first childbirth was also available. The data were obtained from Statistics Denmark (www.statistikbanken.dk), which collects data from the National Danish Birth Registry. The data are anonymous and public available so permission from the national or regional institutional review board was not needed. The database has almost complete inclusion of all children born in Denmark in primary, secondary or tertiary care.

The age specific fertility rate is the ratio between the number of live births by women in a given age group and the number of women in that age group in a certain year. The exact method used by Statistics Denmark to calculate age specific fertility rates differs slightly for data obtained before and after 1973. Before 1973, the numerator of the ratio consists of children born to mothers of a given age at the time of birth, and the denominator is calculated as the midyear population of women with that given age. From 1973, the numerator consists of children born to mothers of a given age at the end of the year and thus not necessarily at the time of birth. The denominator is calculated as the average of the number of women in the given age group at the beginning and at the end of that year. Hence, the new method is cohort based and ensures better agreement between the numerator and denominator. For the younger age groups, the new method results in slightly lower fertility rates while the opposite is the case for the oldest age groups. The numbers are only marginally different when comparing the old versus the new method of calculating age specific fertility rates. The new calculation of the mean population (denominator) takes the seasonal variation in immigration into account. The total fertility at a specific time point is always defined as the sum of all the age specific fertility rates of that year. Cumulative fertility rates were calculated and, together with age specific and total fertility rates, presented graphically to illustrate temporal trends. Furthermore, the contribution of fertility in different age groups to the total fertility was calculated and illustrated as percentages. Medical abortions have been conducted legally in Denmark since 1973. The number and distribution of legal abortions at different age groups was obtained from Statistikbanken in order to estimate age specific healthy pregnancy rates, which were calculated from the sum of live births and induced abortions.

## Results

### Average age at childbirth

**Fig 1:**
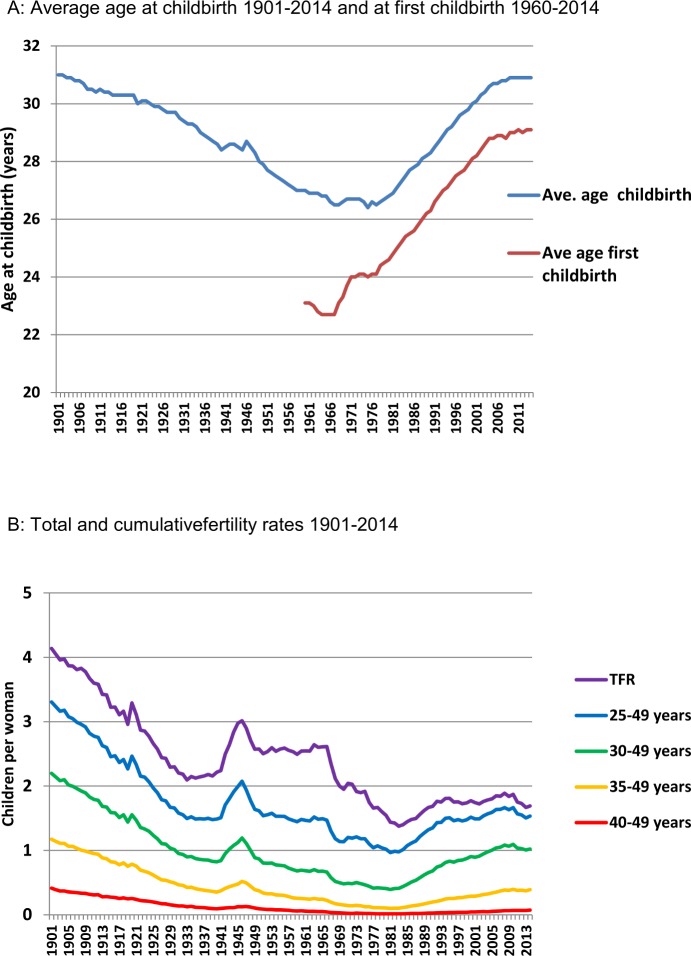
A: Average maternal age at childbirth 1901–2014 and at first childbirth 1960–2014. B: Total fertility rate (TFR) and cumulative fertility rates for selected age groups 1901–2014.

In Denmark, the age of women at childbirth has been recorded consistently since 1900. Interestingly, the average age (~31 years) for women at childbirth was almost identical in 1901 and 2014 (Fig 1A). Information on whether the child was the first born has been available from 1960. Fig 1A shows the steadily increasing average age for first childbirth in Denmark since 1960. In 1960 the average age for women having their first born child was 23.1 years, and maternal age for first childbirth reached 28.9 years in 2004. From 2004 to 2014 the average age for the first born child plateaued around 29 years of age.

### Total fertility rate

**Fig 2:**
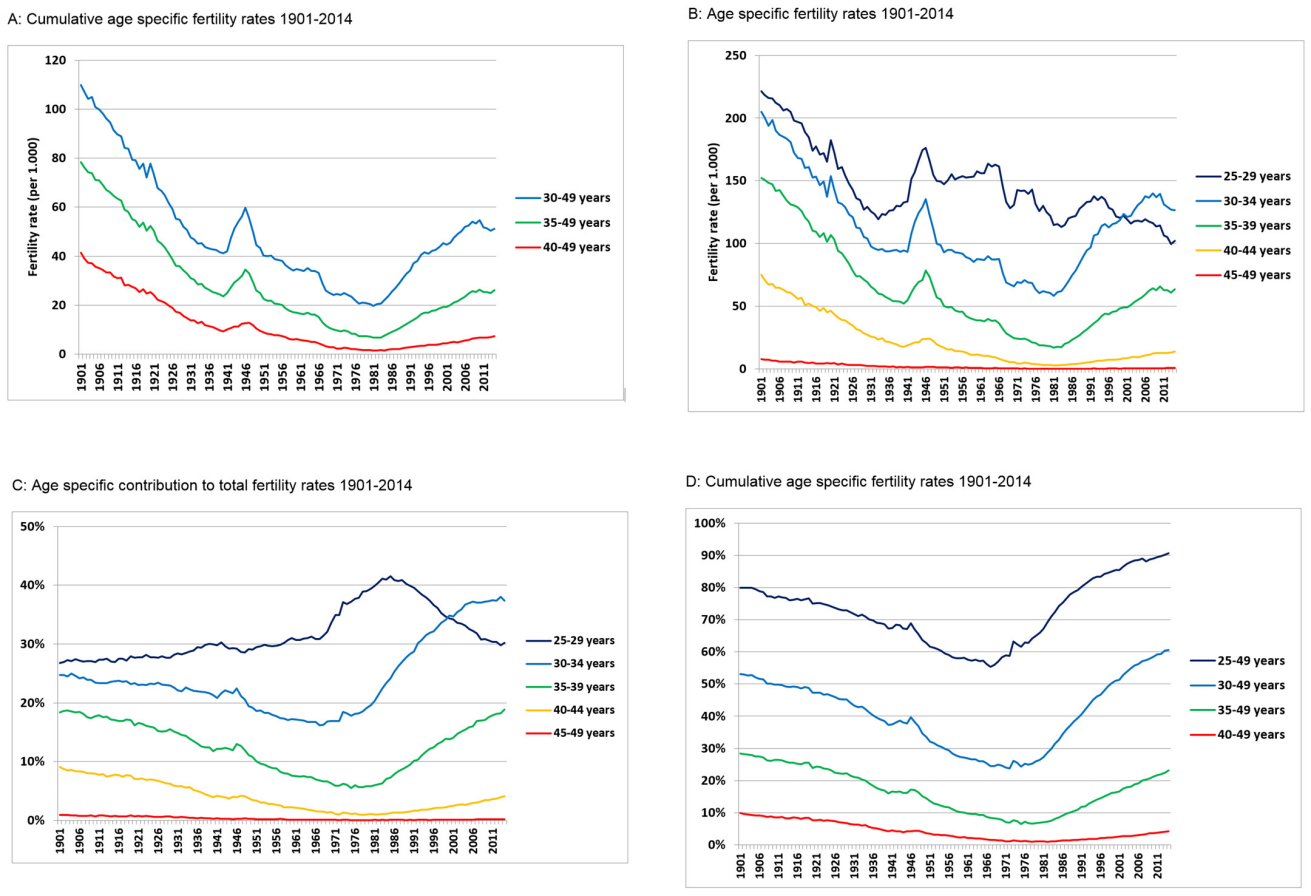
A: Age specific fertility rates 1901–2014. B: Age specific fertility rate 1901–2014. C: Age specific contribution to total fertility rate 1901–2014. D: Cumulative age specific fertility rates 1901–2014.

Total fertility rate (TFR) represents the number of children born to a woman if she were to live to the end of her childbearing years. TFR of Danish women has declined during the 20th century until the 1980s, with two exceptions occurring around the First and Second World Wars when fertility rates increased (Fig 1B). The average woman had more than 4 children in the beginning of the 20th century, whereas the average was only 1.4 children per woman in 1984. In the 1990s, TFR increased to 1.8 and remained around 1.7 and 1.8 children per woman from 1994 to 2014 (Fig 1B). For 100 years ago women had on average more than twice as many children compared with women of today. When stratifying total fertility rates into age groups, it was evident that more than 50% of all children were conceived by women above the age of 30 years from 1901 to 1906 (Fig 2A–2D). Therefore, at the beginning of the century, the average woman gave birth to more than 2 children when she was older than 30 years of age, which is higher than TFR in all age groups in 2014 (TFR equal to 1.8)(Figs 1B and 2D). More than 55% of the children were born by mothers older than 30 years in 2011. The contribution of mothers more than 30 years of age to TFR is for the first time higher since 1901 (Fig 2D). Women above 35 years on average gave birth to 1.2 children in the early 1900s, which exceeds 55% of TFR in 2014 (Fig 2A–2D). In addition, women above the age of 30 years had higher fertility rates from 1901 to 1914 than women above 25 years of age have had since 1949 (Figs 1B and 2C and 2D).

### Age specific fertility rates

Age specific fertility rates change over time and are linked to the temporal changes in TFR (Figs 1B and 2B). The rapid increases in TFR induced by the two World Wars appeared to be smaller in amplitude in the older age groups compared with younger age groups (Fig 2A and 2B). On average, in the age group 40 to 49 years old women 40 children were born per 1000 women in 1901, which rapidly decreased to 9.8 children per 1000 women in 1939. From 1901 to 1939 a decrease in the number of children born per 1000 women was also observed for the 35 to 49 and 30 to 49 year old women (from 77 to 24 and 107 to 41 children per 1000 women, respectively). In 1939, fertility rates for the selected age groups (above 40, above 35, and above 30 years, respectively) corresponded only to 24%, 31% and 38% of the age specific fertility rates in 1901 (Fig 2A). The subsequent increase during the Second World War (1939 to 1945) augmented the difference between age specific fertility rates, which was 28%, 33% and 43% for 40 to 49, 35 to 49 and 30 to 49 year old women, respectively (Fig 2A). After 1945, all age specific fertility rates decreased steadily until they hit an all-time low at the beginning of the 1980s: after which, fertility rates increased again until 2006 (Fig 2A and 2B). In 2001, a fertility rate of 40 children per 1000 women now covered the age group from 30 to 49 year old, which 100 years earlier was achieved by women aged 40 to 49 years (Fig 2B–2D). A decreasing trend from 1900 until the two World Wars was found after stratifying the women into 5 year intervals (Fig 2B). However, the oldest age group comprising the 45 to 49 year old women had low fertility rates in the whole period. The all-time highest annual fertility rate for this age group was 7.7 births per 1000 women in 1901. This number dropped quickly to less than 2 in the 1930s, less than 1 in 1957 and plateaued around 0.5 from 1960 and onwards (Fig 2B). As expected, the relative drop in fertility rate from 1901 to1939 was smaller for the 30 to 34 (50%) and 35 to 39 (65%) year old compared to the 40 to 44 (76%) year old women (Fig 2B and 2C). Similarly, the relative increase in fertility during the Second World War and in the 1980s and onwards was smaller in the older age groups. Interestingly, the fertility rate was higher in the first decade of the 20^th^ century for 35 to 39 year old women compared to the 30 to 34 year old age group at all time points since 1922. The 40 to 44 year old women had also high fertility rates in the first decade of the 20^th^ century, which exceeded the fertility rates of the 35 to 39 year old women from 1949 to 2014(Fig 2B).

The average age at childbirth depend on number of children per mother. Women had more than twice as many children in the beginning of the 20^th^ century compared to the end of the century (Fig 1A and 1B). Interestingly, the average age for childbirth in the early 1900s is almost identical to the 2014 level because they had more than two children after 30 years of age. Actually, the fertility rate of women over 30 years of age in 1901 exceeds the TFR of women in 2014 (Fig 1B). Moreover, it was evident that 10% of the TFR was achieved by mothers giving birth after the age of 40 years in the first decade of the 1900s (Fig 2C and 2D). Only 4% of TFR is achieved by women aged 40 years or more in 2014. On the other hand, it appears that 35–39 year old women are about to reach the same high proportion of TFR as they had in the beginning of the nineteen hundreds with up to 19% of TFR. From 1967 to 2014 age specific fertility for 30 to 34 year old women increased more than all other age groups and accounted for more than 37% of TFR in 2014.

**Fig 3:**
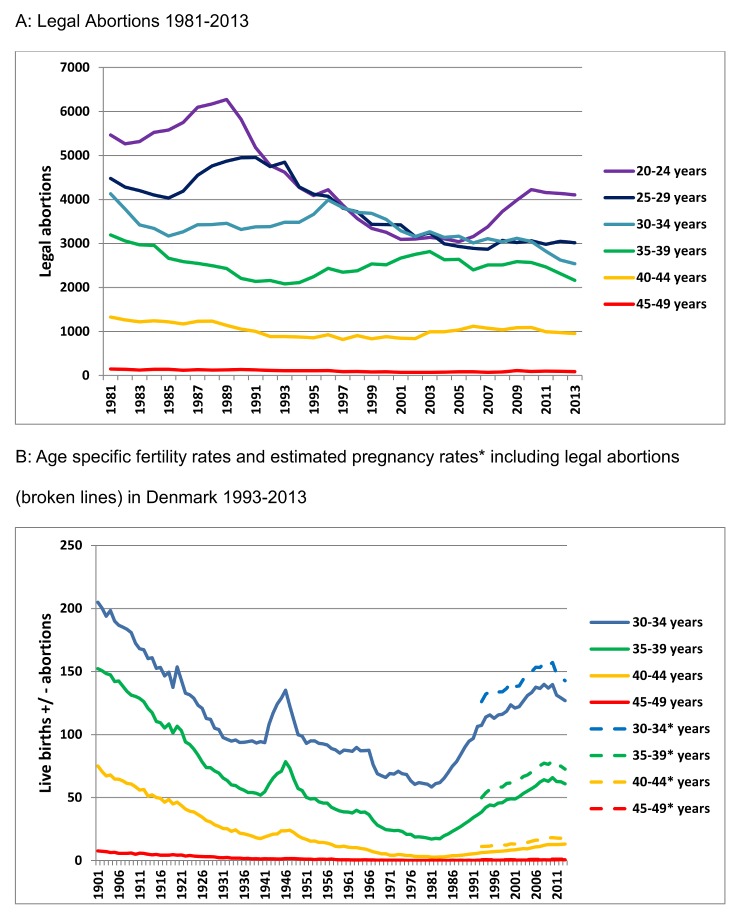
A: Legal Abortions 1981–2013. B: Age specific fertility rates and estimated pregnancy rates including legal abortions (broken lines) 1993–2013.

The number of legal abortions contributes to the changes in TFR from 1973 and onwards. The total number of abortions has decreased by 34% from 1981 to 2013 (Fig 3A), but the number of legal abortions has been relatively stable among women above 30 years of age. If we estimate the number of healthy pregnancies by combining the number of legal abortions and the live birth rate from 1993 to 2013, the age specific healthy pregnancy rates increase on average by 14%, 24%, 57% and 129% for the 30 to34, 35 to39, 40 to44 and 45 to49 year old women respectively (Fig 3B).

## Discussion

This nationwide registry-based investigation of mothers to more than 8 million live births born over a century in Denmark provides novel historical evidence suggesting that women above 30 years are able to sustain fertility rates above replacement level. In the beginning of the 1900s, women gave on average birth to two children after the age of 30 years and more than one child after the age of 35 years. This indicates that chronological age alone did not prevent women in their early or mid-thirties from giving birth to several children. Interestingly, average maternal age at childbirth was almost identical in 1901 and 2014. This highlights that fertility is heavily influenced by demographical, behavioural and social changes. Fertility rates are influenced by multiple factors in addition to age, and the conclusions of this study are compromised by its descriptive nature. Our findings should not be considered as hard evidence but rather an investigation of hypothesised determinants of fertility rates such as increasing maternal age, which requires new well-designed studies to be validated. The use of age specific fertility rates is another limitation of our design, which complicates extrapolation from the whole population to the clinical setting. Age specific fertility rates are influenced by several factors, for instance the number of childbirths per woman and chronological age, and cannot be used as a direct marker for fecundity. Still, if nulliparous women above 30 years of age also are able to sustain fertility rates above two children per woman today, then the observed average age of 29 years to have the firstborn child should not be considered as a major clinical concern with regards to infertility.

Age and fertility of women has been linked for years. Menken and colleagues presented historical data from married couples, which showed a modest 6–14% decrease in fertility until 35 years of age and a steeper decrease after 37 years of age [10]. Similar studies in Mormons [14,15] found a decline in fertility of 10% from women in their twenties until 39 years of age. Thereafter, the decline was steep with a 38% decrease of fertility in the early, and 86% decrease in the late, forties [14,15]. These old marital studies are in line with more recent studies showing that the probability of conception probably does not decline markedly until after the age of 40 years [16,17], which is further supported by our data. The average age at childbirth was high in the first decade of the 1900s and similar temporal trends for TFR and age specific fertility rates as in the present study were also reported in France [11]. Interestingly, their data also included age at first childbirth from 1900 to 2006. The age at first childbirth was at an all-time high in 2006, but age at first childbirth has been high previously in France (1.6 years lower than at present) without causing a decline in TFR [11]. Several studies have investigated the impact of age for the declining fertility in women. A cohort study [18] investigating couples with proven fertility found a significant shorter time to pregnancy (TTP) for women below 27 years of age compared to 33 year old women. Thereby indicating an earlier decline in fertility compared with our findings and the studies In Mormons and marital couples. The results from this cohort study was severely compromised by a short median TTP of only two months, which may explain why the results differed from a large multicentre studies investigating 942 fertile couples from four different cities in Europe. Their study found no difference in chronological age between women with a 1–6 months TTP (29.2 years) and a TTP >6 months (29.1 years) [19]. Instead women with TTP > 6 months had a significant higher frequency of previous urogenital disorders, lower frequency of sexual intercourse, irregular bleeding pattern and lower educational level, which all are known to be important for fertility. Another prospective study [20] found a 40% reduced probability of clinical pregnancy in the 35–39 year old group compared to 19–26 year old women. The impact of maternal age was also limited in a prospective study investigating 295 fertility planners [21]. The proportion of couples conceiving differed significantly when stratified into two groups based on normal sperm concentration, while female age, frequency of female reproductive diseases, length of menstrual cycle and smoking were identical between the groups, which highlights that fertility also depends on male reproductive health.

Two elegant studies [22,23] bypassing the male partner found that age above 35 years was a determining factor in women married to azoospermic men who received donor insemination. Conception was lower in women above 30 years of age, however, the difference in conception rates was barely detectable and was only statistically significant after 12 menstrual cycles. Noteworthy, there was no difference in live birth rate when comparing all age groups after 1–4 cycles or after 24 cycles [23]. Most studies report a steep decline in fertility in their late thirties between 35–38 years rather than after 30 years, although the results depend on the reproductive health of the cohort and the endpoints used especially conception versus live birth. It is difficult to extrapolate results from fertility clinics evaluating chronological age as a determinant for fertility potential during ART to the normal population because couples at the clinics have more reproductive problems, medication and co-morbidities. Still, the best evidence for causality in age dependent impaired fertility comes from oocyte donation studies [24–26]. These studies conclude that impaired fertility in older women (>40 years) is largely a result of degenerative changes within the aging oocyte rather than other senescent changes, because fertility in older women can be reversed using oocyte donation from younger women. Heritability has a major influence on ovarian aging, and different strategies and approaches have been attempted to make a proper ovarian aging evaluation [1]. Current use of diagnostic tools such as serum AMH, Inhibins, sex hormones, FSH, gonadal markers and ovarian imaging has improved prediction of biological ovarian age, which may be a better predictor of fertility than chronological age [27,28].

In conclusion, this study provides historical evidence for women above thirty years of age to produce enough children to sustain replacement level in most developed countries of today. Clinical research should prioritize to identify factors that influence or can be used to assess accelerated biological aging rather than focus on chronological age alone, which appears to be of less importance until after 35 years of age.
